# Supplementary material for: What Tweets and YouTube comments have in common? Sentiment and graph analysis on data related to US elections 2020
Source: PLoS One. 2023 Jan 31;18(1):e0270542. doi: 10.1371/journal.pone.0270542 (PMC9888715; doi:10.1371/journal.pone.0270542)
Supplement: S1 Appendix — (PDF) [file pone.0270542.s001.pdf]

7 List of all Hashtags

In this Section, we list of the most popular retrieved hashtags on which we based our dataset along with the tweets count within which they were contained, in Table 5.

6.

Table 5. The list of 20 most popular hashtags in our dataset. The entire list of hashtags contain 585.486 unique entries.

Hashtag	Tweets count
#vote	7.196.981
#trump2020	3.913.969
#election2020	3.535.323
#biden	1.569.981
#trump	959.048
#bidenharris2020	866.120
#debate2020	855.543
#votebluetosaveamerica	837.089
#maga	697.381
#trumphascovid	581.456
#donaldtrump	555.251
#electionday	530.188
#covid19	394.538
#2020election	370.900
#voteearly	316.226
#bluewave2020	307.853
#kag	295.178
#elections2020	270.224
#vote2020	261.161
#electionnight	255.496
Total	24.569.906

Table 6. The list of Twitter hashtags that was used in order to collect 2020 US Election dataset.

Hashtag	Tweets count
#vote	7.196.981
#trump2020	3.913.969
#election2020	3.535.323
#biden	1.569.981
#debate2020	855.543
#votebluetosaveamerica	837.089
#trumphascovid	581.456
#donaldtrump	555.251
#2020election	370.900
#bluewave2020	307.853
#elections2020	270.224
#uselection2020	229.675
#2020elections	190.637
#votetrumpout	164.256
#uselection	87.827
#elections	50.158
#november3rd	36.785
#mypresident	34.591
#novemberiscoming	33.585
#uselections	17.691
#getyourassoutandvote	2.431
#2020usaelection	2
Total	20.842.208
