# Supplementary material for: What Tweets and YouTube comments have in common? Sentiment and graph analysis on data related to US elections 2020
Source: PLoS One. 2023 Jan 31;18(1):e0270542. doi: 10.1371/journal.pone.0270542 (PMC9888715; doi:10.1371/journal.pone.0270542)
Supplement: Attachment — Submitted filename: response_to_reviewers_round2.pdf [file pone.0270542.s004.pdf]

PONE-D-20-37937R1
What Tweets and YouTube comments have in common? Sentiment and Graph analysis on data related to US Elections 2020
Dr. Despoina Antonakaki

The original review letter:

Dear Dr. Antonakaki,

Thank you for submitting your manuscript entitled "What Tweets and YouTube comments have in common? Sentiment and Graph analysis on data related to US Elections 2020" to PLOS ONE. Your manuscript files have been checked in-house but before we can proceed we need you to address the following issues:

(1) Can you please clarify the following points?:

1.1) Whether authors had access to any identifying information for individual Twitter users.

Yes, we had access to the usernames, and we did not anonymize them, we inserted data in a database. However, there is no reference to usernames or any other identifying information for the users in the manuscript or the publicly available dataset, except the news channels or public figures involved in the online discourse, in the plots.

1.2) If the dataset was aggregated or anonymized before analysis.

The dataset was not anonymized and was inserted in a mongo DB. There is no reference to usernames or any other identifying information for the users in the manuscript.

1.3) If a data protection or other ethics committee provided oversight of the data collection process.

We consulted our Data Protection Officer (DPO) for the usage of this dataset.

Thank you for your attention to these queries.

(2) Thank you for providing additional details regarding the restrictions on data sharing. We note that data from https://zenodo.org/record/4618233#.YGGJU2Qzada are available upon request. In line with our goal of ensuring long-term data availability to all interested researchers, PLOS' Data Policy states that authors cannot be the sole named individuals responsible for ensuring data access (http://journals.plos.org/plosone/s/data-availability#loc-acceptable-data-sharing-methods).

Before we proceed with your manuscript, please address the following prompts:

----

a) If there are no ethical or legal restrictions on sharing your Zenodo dataset publicly, please make the dataset public.

The dataset is now public in Zenodo, available at the same link.

----

b) If the dataset will be public upon acceptance of the manuscript for publication, please state so explicitly.

The dataset is already publicly available in Zenodo.

----

c) Please also provide information on how the underlying data can be obtained. Alternatively, please provide contact information to which data requests may be sent. This contact should not be one of the authors of the paper.

We provide information as a separate file: how_to_obtain_dataset.pdf submitted with the manuscript.

----

e) Please confirm that the authors did not have any special access to the data that other qualified researchers cannot obtain. Please note that PLOS will not consider manuscripts whose conclusions depend solely on the analysis of proprietary data (https://journals.plos.org/plosone/s/data-availability#loc-unacceptable-data-access-restrictions).

All the data required for this analysis is available through the Twitter API and YouTube API.

We will update your Data Availability statement on your behalf to reflect the information you provide.

----

(3) Please ensure that you refer to Figure 3 and 4 in your text as, if accepted, production will need this reference to link the reader to the figure.

Figures 3, 4 are not referenced in the manuscript because that part of the text has been removed in the revised version. So we have removed Figures 3,4 from the submission as well. Consequently, we have renamed all the subsequent figures to the correct numbers and the filenames submitted.

----

(4) Please include a legend for figure 3 and figure 4.

Figures 3, 4 have removed.

Your manuscript has been returned to your account. Please log on to PLOS Editorial Manager at https://www.editorialmanager.com/pone/ to access your manuscript.

Your manuscript can be found in the "Revisions Sent Back to the Author" link under the New Submissions menu. After you have made the changes requested above, please be sure to view and approve the revised PDF after rebuilding the PDF to complete the resubmission process.

Please note that these changes have been requested to comply with submission guidelines and your manuscript will *not* be sent to review until you have fully adhered to our requests. Once your paper has been seen by an Editor we may return it to you for further information or amendments.

We ask that you address this request within 21 days. If you require additional time, please email the journal office. We are happy to grant extensions of up to one month past this due date. If we have not heard from you within 21 days, your manuscript will be withdrawn from Editorial Manager.

Thank you for submitting your work to PLOS ONE.

Kind regards,

Orsolya Voros, PLOS ONE
