# Supplementary material for: What Tweets and YouTube comments have in common? Sentiment and graph analysis on data related to US elections 2020
Source: PLoS One. 2023 Jan 31;18(1):e0270542. doi: 10.1371/journal.pone.0270542 (PMC9888715; doi:10.1371/journal.pone.0270542)
Supplement: Attachment — Submitted filename: Response_to_the_reviewers_third_round.pdf [file pone.0270542.s005.pdf]

Response to the reviewers

Review comment 1) The most significant weaknesses in this paper are its lack of clear contributions to the field and the (relatedly) limited connection it has with prior work studying political discourse in lead-up to elections on social media. For example, there's a huge body of literature on Twitter and politics in general [1], and the 2016 US presidential election specifically [2-3], so how does that work inform what we might expect to find in Twitter and YouTube during the 2020 election? Are any of the results identified in the four months leading up to Election Day in 2020 surprising given what we know about 2016? Even for 2020, other work has been done on YouTube and sentiment (e.g., Singh and Sikka [4]), so how consistent are these results with those from other papers in this area?

Abstract, Introduction and Background work have been reformed and as we have included a lot of cross-platform studies.
We have also included the papers mentioned in the review comment, among other new background work that we have added. [1] is referenced in subsection: "*Political content analysis on Twitter*", of section "*Background*". [2] is referenced in the first paragraph of "*background*", [3] is referenced in the first paragraph of subsection "*Political content analysis on Twitte*r" in "*Background*" and [4] is referenced in the first paragraph of subsection "*Content analysis on YouTube"* in "*Background".*

Regarding the results in the four months leading to election day in 2020 from our analysis, cannot correlate with previous work done for 2016. The nature of the two analyses are different and the dataset in the previous studies (in 2016) seem to be limited , in terms of number of tweets as well as covering multiple social media. In these terms we cannot perform a comparison between the two studies. More specifically in [1 BARBERÁ, P., CASAS et al.] they use a dataset of tweets sent by the members of the 113th House and Senate of the US Congress (2013–14), while our dataset contains tweets from the popular hashtags available, which means they could be sent by users (including electorate) and potentially members of the House and Senate. Additionally, they do not include data from other social networks (like YouTube in our study) and the pipeline of the analysis is completely different (e.g. they apply probabilistic model LDA, and we are not applying topic analysis at all).

The same applies for the related studies in 2020 (e.g., Singh and Sikka [4]). Specifically, they are applying an analysis on 200 YouTube comments, while our analysis includes 20M tweets and the comments of 29K YouTube videos. The nature of the two analyses is different in many perspectives, since we apply a comparative analysis between the two social networks (Twitter and Youtube) and sentiment analysis is just a step of the pipeline necessary towards this goal, while authors in [4] are solely focusing on the sentiment analysis of the YouTube comments.

Finally, regarding [2] what they study is more close to our analysis, except that they are comparing Twitter and Facebook, while our analysis focuses on Twitter and YouTube. Also, they focus the emotional frames while we demonstrate how the communities correlate between Twitter and YouTube.

[The last two paragraphs are not included in the manuscript, since it is a response to the review and out of the context of the study].

-Review comment 2) While the paper's related work section does identify a number of related efforts in this area, more needs to be done to explain what *important* questions this paper answers that have not already been answered. That is, we need to know not just how this paper investigates a slightly different context than the other papers, but how those other papers inform this work. Does this work contradict existing results or expectations? Does the existing body of work contradict itself, and this paper resolves this contradiction? These are crucial questions that need to be answered to connect this work to a particular community and demonstrate what the most important result in this paper really is.
Related to above, the paper lacks a clear set of hypotheses about what we might expect in this context. This work is not exploratory given the volume of work in this area, and providing a set of hypotheses based on the literature would help us understand the important results in this work.

We have reformed the paper including the abstract, the introduction, and the background work, highlighting our reshaped contributions that focus on the correlation and interactions between Twitter and YouTube communities, which actually is our novelty.

-Review comment 3) Beyond this contextualization, data collection and validity of the data collected in this effort are unclear. How exactly was this dataset collected from Twitter? I understand the API was used, but was the streaming API used or the retrospective API? If the collection was built around particular keywords, what were those keywords, and how were they selected? The paper mentions a list of hashtags, but it appears this list is extracted from the collected dataset, not a set of tags used to develop the dataset. We need additional detail about how this dataset was collected to assess what is missing. For example, popular hashtags like #StopTheSteal, #HidingBiden, or other partisan hashtags do not appear in this data.
To address this issue, the paper should clearly explain exactly how the data was collected and why this timeframe is reasonable.

We address this comment in the “Dataset” section, where Twitter and YouTube dataset collection procedure is described.
Considering the hashtags used for our analysis, we only show in the manuscript the list of 20 most popular hashtags in our dataset. The entire list contains 585.486 unique entries of hashtags, which is too long to be added to the manuscript and is included in the submission as a separate file (all_hashtags.txt) which contains all the HTs used and the number of tweets corresponding in each HT that we have in our dataset.

We added in the manuscript the following paragraph:

We considered that this date was a reasonable starting point for collecting our dataset since the amount of tweets in the corresponding hashtags we collect begin to accumulate a significant number, as well as the semantic of the content, started to be more relevant to the conversation related to the elections.
Regarding the completeness of the content covered by our dataset, we acknowledge the fact that additional minor hashtags may exist during that period that were not crawled and included in our corpus. We consider our dataset a complete online discourse since we got the majority of the hashtags available in that period before the elections. Additionally, there was an overlap between the hashtags on the election discourse and these hashtags were cross referenced in the tweets. For example, some tweets included the popular hashtags (\#Vote) and the minor hashtags were also mentioned in the same tweet. This tweet is included in our dataset because of \#Vote. Additionally, we included only the hashtags that we general and not in favor of a particular candidate. We try to include only two hashtags that are in favor of each candidate, keep a balance between them and not introduce a bias towards one

of them. Finally, the main amount of the political conversation was gathered in the popular hashtags and the rest of them do not contain a significant amount of tweets. In the appendix, in figure \ref{table:allhashtags} we show the list of 20 most popular hashtags in our dataset. The entire list contains 585.486 unique entries of hashtags, which is too long to be added to the manuscript.

We also included in our submission the whole list of HTs crawled for this dataset. [filename : all_hashtags.txt]

-Review comment 4) The paper also claims to present an analysis of sentiment per US state, but no information is provided about *how* this analysis maps messages to US states. Geolocation in social media is a known and open problem, and the inclusion of this analysis necessitates a description of how this geolocation is performed.

This comment is addressed in the “Sentiment Analysis” section.

As it appears in the manuscript:

In order to identify the location of the user accounts, we extract this information from the corresponding field named 'location' in the Twitter user object. Additionally, we append this information with the location field in the tweet object of the Twitter API. We acknowledge the fact that the first field may be not updated by the user or the second field could be missing because the user did not approve to share the location. This means that the location for many users could be missing. Nevertheless, this is the closest information we have from Twitter about the user location.} We notice the daily fluctuations for every state per entity (blue is the entity 'Biden' and red is for 'Trump'). The juxtaposition of the time series in the form resembling an EEG makes it easier to discern localized events from nation-wide Twitter traffic.
The list of state abbreviations can be found here: \cite{states_abbr}.

-Review comment 5) The analysis of retweet graphs are primarily qualitative and lack a compelling frame. What did we expect to see here? How do we know the differences observed between the two candidates is meaningful?

As written also in the manuscript:

The retweet graphs show the volume of the tweets and the relation between the nodes which represent the users. This apposition of the graphs through the whole pre-elections period, demonstrates how the volume of the communication and interaction between the involved users evolves. The results were expected as we see the density of graph increasing, which means the volume of the tweets increases, and the discourse is getting more intense, as we approach the period close to the elections.

The discussion around each candidate evolves through time as well. The corresponding hashtag for each candidate is mentioned as long as the electorate references them in the discourse. Of course, this does not necessarily means a preference towards Trump or Biden. As mentioned, as well in section \ref{dataset}, this analysis does not focus on the prediction of the preference of the electorate or the outcome of the elections, we expect to see how the users engage in the online conversation. As we get closer to the election date, we notice some components being formed in the graph that show the conversations about each candidate and the conversation about both of them in figure 11 [or in \ref{retweet_graph}] in the last bottom sub-graph (f).

Additionally, we see two main graph components throughout the whole period that change colour according to the daily events. For example, in the button left subgraph (e) we notice that the colour is red in both components while the button right figure (f) one is painted blue (from J. Biden) . This happens because the left graph highlights the fact that Trump was infected with COVID-19 on 2/10, and the conversation about Trump was more intense and increased in terms of tweets. Also in the middle right plot we show the day after the debate, which mixed red-blue colour makes sense if we consider the conversations this particular debate initiated. The colours as expected seem to resume back to normal on the last bottom right (3/11, which correspond to the main two components blue and red, one for each candidate, since on this date none of the candidates seem to attract any peculiar attention.

-Review comment 6) Sentiment analysis in Vader is not specifically built for social media; the paper should spend some effort demonstrating its assessment of sentiment towards the presidential candidates is valid.

As justified in the text as well :

There is a plethora of previous studies as mentioned in section \ref{Background} that analyze the sentiment in Twitter. Vader is broadly used in this domain as shown in \cite{zahoor2020TWSent, elbagir2019twitter, pano2020Complete, ramteke2016election, shelar2018sentiment, park2018sentiment, mustaqim2020twitter, bose2021survey, al2020suspicious, yaqub2020tweeting, alharbi2019twitter}.

-Review comment 7) More detail is needed for entity extraction. Is the search for trump/biden a direct token match or a substring match? E.g., would it match a tweet with #TeachersForBiden? Also, for tweets with tags like #VoteBlue, they may be specific to Biden but not directly mention him; how does that potentiality impact analyses?

As described in section "The Entities and their sentiment" of the manuscript, the entities of J. Biden and D. Trump were identified with a computed set of keywords (including the hashtags and enriched with the candidates' names and the parties they are representing). The selected keywords are shown in table ref{table:allentities} [Table 2: The complete list of all entities with the corresponding keywords that were used for each one.] . For example hashtag \#VoteBlue, as a text does not contain the word Biden, but is associated with J. Biden campaign, and we manage to recognize it because of our keywords shown in table 2.

For this reason, we use as keywords the hashtags correlated with each candidate. This approach increases the accuracy of the association of a particular tweet/user with the described entities.
\The keywords are being searched in lower case in order to avoid any misspelling and user upper-lower case writing style.

-Review comment 8) In general, node-edge diagrams are not particularly useful visualizations. Additional effort is needed to describe what is interesting about the communities in these figures, what size means, why certain nodes are labeled and others note, etc.

This is explained in detail in the manuscript in section 5 Linking Twitter and YouTube data:

Fig17.eps illustrates the 3-core YouTube comment graph that represents the relation of which channel has commented on which channel. Different colored areas (purple, light grey, green blue) in the graph represent different communities that are formed between the YouTube channels produced by the Louvain algorithm. For example purple indicates the community between the "Blaze TV", "The Officer Tatum" and the " Fox News". However " Fox News" is also between the purple and the grey which shows the next community (formed by "Fox News" and "Donald J Trump"). These communities do not necessarily correspond to a real life community are a result of the Louvain algorithm of Gephi.
Regarding the labels, in this figure we also show the 13 most popular channels after running PageRank.

Similarly, in plot 18 we show the 3-core Retweet graph that represents the relation which users have retweeted. In the graph we notice seven colored areas of different size, indicating different communities, that as in plot 17, do not necessarily correspond to a real life community. Also the text labels shown, for example "MaryLTrump" and "DrDenaGrayson" seem to be close, which again cannot be translated to semantic information. It is considered coincidental to be close but not coincidental to be in the same color-area (community).

In both plots (node diagrams 17.eps-18.eps) it is not possible to keep readable names of each particular node, through the high density of nodes. The nodes in Fig18.eps are the users in Twitter who have posted the tweets included in this plot and similarly the nodes in Fig17.eps are the users of YouTube which are channels. In order to highlight only the important nodes we manage to provide the names for most significant nodes based on the PageRank score.

For illustration purposes nodes in both plots (17,18) with high degree are shaped like in circles (lightly highlighted circle-like scheme) in this plot, but are variables in Gephi that unfortunately, cannot be explained semantically in this context and should be ignored.

-Review comment 9) The paper is missing a "Data Availability Statement" in the text.

The dataset is available through zenodo and this was already described in subsection: “*How to obtain the dataset*”, which has been moved to subsection: “*Data Availability”* subsection located in the dataset section.

https://zenodo.org/record/4618233#.YGGJU2Qzada
